# Supplementary material for: Reconfigurable structured light generation in a multicore fibre amplifier
Source: Nat Commun. 2020 Aug 10;11:3986. doi: 10.1038/s41467-020-17809-x (PMC7417554; doi:10.1038/s41467-020-17809-x)
Supplement: Supplementary file 1 — Supplementary Information [file 41467_2020_17809_MOESM1_ESM.pdf]

Supplementary Information for "Reconfigurable structured light generation in a multicore fibre amplifier"

DI LIN,[1*] JOEL CARPENTER,[2] YUTONG FENG,[1] SAURABH JAIN,[1] YONGMIN JUNG,[1] YUJUN FENG,[1] MICHALIS N. ZERVAS,[1] DAVID J. RICHARDSON[1]

[1]*Optoelectronics Research Centre, University of Southampton, Southampton, SO17 1BJ, UK*
[2]*School of Information Technology and Electrical Engineering, The University of Queensland, Brisbane, Queensland 4072, Australia*
**di.lin@soton.ac.uk*

Supplementary Note 1. Numerical calculation of beam combining efficiency

In our experiment, the output beam of the MCF was first magnified by a factor of 10 through a 4F imaging system to ensure that the separation distance of the beamlets can be matched to the lens pitch of an MLA at its input plane (see **Supplementary Figure 1**). Here we assume that the N beamlets (N = 6 in our experiments) are identical linearly polarized Gaussian beams with a beam waist of w_0 (w_0 = 28 μm) and an initial phase φ_j (j = 1 to N) and that they are evenly distributed on a circle at positions (x_{0j}, y_{0j}). The electric field distribution at the input plane can be expressed as

$$E_0(x,y)=\sum_{j=1}^{N}a_j\cdot\exp\left(-\frac{\left(x-x_{0j}\right)^2+\left(y-y_{0j}\right)^2}{w_0^2}+i\varphi_j\right) \tag{1}$$

where $x_{0j}=r\cdot\cos(\theta_j)$, $y_{0j}=r\cdot\sin(\theta_j)$, $\theta_j=(2j-1)\pi/N$, and a_j is the amplitude of the Gaussian beam. **Supplementary Figure 1**(b) illustrates a schematic of our optical system for coherent beam combination with a tiled-aperture configuration. The input beam, $E_0(x,y)$, first propagates towards the front surface of the MLA and, assuming Gaussian beam propagation, the electric field $E_{11}(x,y)$ at the input to the MLA can be described as follows:

$$E_{11}(x,y)=\sum_{j=1}^{N=6}a_j\cdot\exp\left\{-\left[i\frac{k}{2R(z)}+\frac{1}{w^2(z)}\right]\left[(x-x_{0j})^2+(y-y_{0j})^2\right]+i\varphi_j\right\} \tag{2}$$

where k is the wavenumber ($k=2\pi/\lambda$, where λ is the wavelength), $R(z)=(z^2+z_r^2)/z$ is the radius of curvature, $z_r=\pi w_0^2/\lambda$ is the Rayleigh range and $w(z)=w_0(1+(z/z_r)^2)^{1/2}$ is the radius of each beamlet at the propagation distance z. The MLA was fabricated on a fused silica substrate with a dimension of 20×20 mm, which is much larger than the size of the beamlet array and the transmission function can be described as:

$$T(x,y)=T_0\begin{cases}\sum_{j=1}^{N=6}\exp\left[-i\frac{k}{2f_1}\left[\left(x-x_{0j}\right)^2+\left(y-y_{0j}\right)^2\right]\right]\cdot circ\left(x-x_{0j},y-x_{0j},D\right), & \sqrt{\left(x-x_{0j}\right)^2+\left(y-y_{0j}\right)^2}<D\\ 1, & otherwise\end{cases} \tag{3}$$

where T_0 is the transmission amplitude of the MLA, which is estimated to be ~0.965 taking into account the Fresnel reflections from both uncoated surfaces, D is the diameter of each microlens (D = 500 μm) and *circ*() is the circular function defined by

$$circ(x,y,D)=\begin{cases}1, & \sqrt{x^2+y^2}<D\\ 0, & otherwise\end{cases} \tag{4}$$

The electric field behind the MLA can be described as follows:

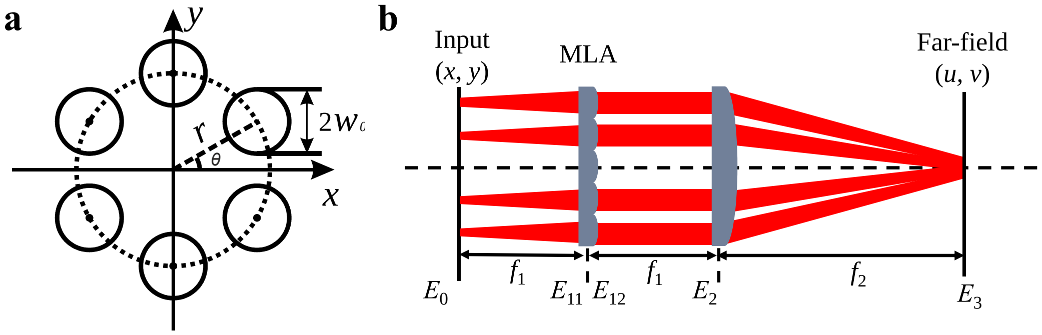

Supplementary Figure 1. (a) Schematic of the beamlet array with N = 6 identical fundamental Gaussian beams and (b) optical system for tiled aperture beam combination.

$$E_{12}(x,y) = T(x,y) \cdot E_{11}(x,y) \tag{5}$$

The MLA is used to collimate the beamlet array and to improve the near-field filling factor and beam combining efficiency. After the MLA, the electric field distribution at the propagation distance $z = f_1$ can be derived with the Fresnel diffraction integral as

$$E_2(x,y) = \mathcal{F}^{-1}\left\{\mathcal{F}\left\{E_{12}(x,y)\right\} H\left(f_x, f_y\right)\right\} \tag{6}$$

where $\mathcal{F}\{\}$ and $\mathcal{F}^{-1}\{\}$ denotes the two dimensional Fourier transformation and inverse Fourier transformation, respectively and the transfer function H is given by

$$H\left(f_x, f_y\right) = \exp\left[ikz - i\pi\lambda z\left(f_x^2 + f_y^2\right)\right] \tag{7}$$

The electric field on the back focal plane of the lens (f_2 = 200 mm) can be approximated by the Fraunhofer diffraction integral as

$$E_3(u,v) = \frac{\exp\left(i\frac{k}{2f_2}\left(u^2+v^2\right)\right)}{i\lambda f_2} \mathcal{F}\left\{E_2(x,y)\right\} \tag{8}$$

(u, v) is the coordinate in the far-field plane (at the back focal plane of the lens), and the intensity distribution is given by

$$I_3(u,v) = \frac{1}{\left(\lambda f_2\right)^2}\left|\mathcal{F}\left\{E_2(x,y)\right\}\right|^2 \tag{9}$$

Supplementary Figure 2 shows the amplitudes ($|E_3|$) of the electric field distributions of various structured light beams in the far-field plane **Supplementary Figure 2**(a)-(c) and (f) show the amplitudes of scalar LP modes as shown in Figure 4 and **Supplementary Figure 2**(d)-(f) represents the amplitude of the coherently combined beam when the initial phase of the beamlets has a total linear chirp of 2π, 4π and 6π, respectively. Due to the limited number of beamlets (N = 6) in our experiment, a relatively small phase difference between the adjacent beamlets is needed to form a ring-shaped beam and it should less than π/2 in order to get a ring-shaped OAM beam as shown in **Supplementary Figure 2**(d). The beam combining efficiency is defined as the ratio of the power contained within the circular (or rectangular) white lines to the total power in the beam and it varies for different structured light beams. Our calculations show that the fundamental Gaussian beam (**Supplementary Figure 2** (a)) has the lowest predicted combination efficiency of ~46 % and the LP_{31} higher order beam (**Supplementary Figure 2** (f)) has the highest combining efficiency of ~90 %. The first order OAM beam (**Supplementary Figure 2** (d)) has a predicted combination efficiency of ~60 %. The higher combining efficiency of the higher-order modes can be attributed to the greater similarity of the electric field distribution of these combined beams to the input beamlet array, resulting in a lesser side-lobe power contribution.

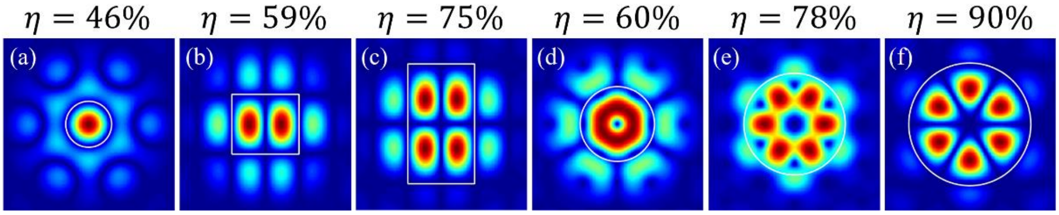

Supplementary Figure 2. The calculated electric field distributions $|E_3|$ and beam combining efficiencies of different structured light beams.

Supplementary Note 2. Two-dimensional correlation coefficient

We quantify the degree of agreement between the measured output beam intensity distribution and the theoretical prediction by calculating the two-dimensional correlation coefficient (C) between the measured intensity distribution ($I_{\mathrm{m}}(x, y)$) and the target intensity distribution ($I_{\mathrm{t}}(x, y)$) of each mode [1]:

$$C = \left| \frac{\iint \Delta I_{\mathrm{m}}(x,y)\Delta I_{\mathrm{t}}(x,y)dxdy}{\sqrt{\iint \Delta I_{\mathrm{m}}(x,y)^2 dxdy \iint \Delta I_{\mathrm{t}}(x,y)^2 dxdy}} \right| \quad (10)$$

where $\Delta I_j(x,y) = I_j(x,y) - \bar{I}_j$ with j = m, t and $\bar{I}_j$ is the respective mean value of the intensity distributions of I_{m} and I_{t}. A correlation coefficient of 1 indicates that the measured beam profile fits perfectly with the target beam and a correlation coefficient of zero indicates no correlation. For the scalar LP modes in Figure 4, the two-dimensional correlation coefficients were calculated to be 0.9826, 0.9773, 0.9712 and 0.9319 for LP_{01}, LP_{11}, LP_{21} and LP_{31}, respectively and the correlation coefficient for the CV beams shown in Figure 5 was calculated to be ~0.9701.

Supplementary Note 3. Normalized merit value as a function of optimization time

The system takes about 10-15 minutes to reach the optimized output beam in our experiment. **Supplementary Figure 3** plots one example of the merit value as a function of optimization time when converting a Gaussian-shaped beam into a doughnut-shaped CV beam.

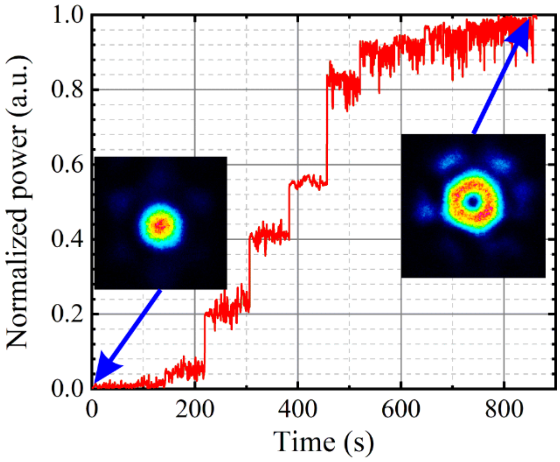

Supplementary Figure 3. Normalized merit value vs optimisation time.

Supplementary Note 4. Key factors affecting the beam combination efficiency

There are several factors that can affect the beam combination efficiency and we numerically simulated and analyzed the effect of the two main factors; i) inappropriately collimated beams (longitudinal misalignment) and ii) lateral offset of the optical components from the optical axis (lateral misalignment).

Supplementary Figure 4 (a) shows examples of the amplitude of the electric field (|E|) distribution of the coherently combined doughnut-shaped OV beam when the position of the beam waist of the magnified beamlets deviates from the front focal plane of the MLA (z is the distance between the beam waist and the MLA). It can be seen that a defocus by 15 % can significantly increase the side lobe intensity and it can be understood that the divergent beamlets in the near-field result in increased electric field components with higher spatial frequencies in the far-field, and hence a reduced optical intensity within the desired central lobe of the coherently combined beam. **Supplementary Figure 4**(b) shows the one-dimensional amplitude of the electric field along the x-position when z/f = 0.85 and z/f = 1.0, which clearly shows that the defocused beam waist can lead to lower amplitude within the central lobe and higher side lob intensity than the optimized beamlet. **Supplementary Figure 4**(c) shows the calculated beam combination efficiency of the doughnut-shaped OV beams as a function of position of the beam waist of the magnified beamlets. It can be seen that a defocus by ~15 % (Δz = 1.8 mm, given that the

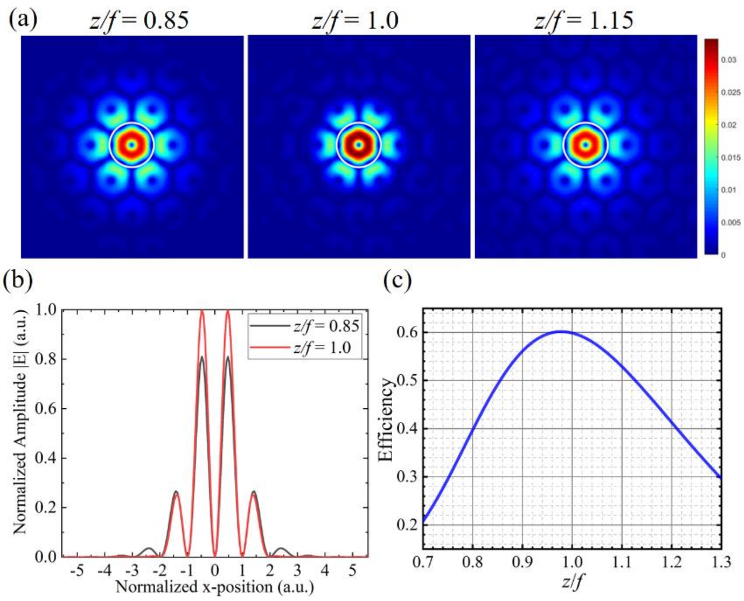

Supplementary Figure 4. (a) Far-field amplitude of electric field distributions (|E|) under different collimation situations, (b) one-dimensional normalized amplitude of electric field along the x-position when z/f = 0.85 and z/f = 1.0 and (c) calculated beam combination efficiency in the central lobe as a function of z/f

focal length of the MLA is 12 mm) can reduce the combination efficiency below 50 %. Note that the position of the maximum combination efficiency shifts a little towards $z < f$ in our case because the beam size of each beamlet (d = 426 μm when $z = f$) on the front plane of the MLA is close to the clear aperture of the microlens (D = 500 μm), resulting in a non-negligible diffraction loss due to the limited clear aperture of the microlens when the beam size increases with the increase of z.

The other factor that has a significant impact on the beam combination efficiency is the lateral offset of the optical components in our coherent beam combination setup (i.e. lateral offset between MCF, lenses and MLA). Any lateral misalignment of the optical components can lead to a shifted location of the coherent combined beam in the far-field and reduced beam combination efficiency. Here, to gain some insight, we analyzed the effect of positional drift by simulating the far-field intensity profiles of the coherently combined OV beams when the position of the MCF is moved along the x-direction. As shown in **Supplementary Figure 5**(a), a 1.5 μm positional drift can result in severe beam distortion and an eventual decrease in the beam combining efficiency. Note that the optical power of the main lobe gradually shifts to the adjacent left side lobe on applying a lateral offset and most of the optical power can be shifted to the left side lobe with a 4.35 μm positional drift. The optical power can be further shifted to the next left side lobe as the offset increases further and it shows periodic behavior with a 4.35 μm drift as shown in **Supplementary Figure 5**(b). The laterally shifted doughnut-shaped beam always accompanies a reduced intensity due to the increased diffraction loss caused by the increased truncation of each beamlet by the corresponded microlens with a limited clear aperture size. **Supplementary Figure 5**(c) shows the calculated beam combination efficiency of the ideal OV beams with different orders of shift. The efficiency drastically decreases from ~60 % to ~15 % when the position of MCF drifts ~21.75 μm along the x-position.

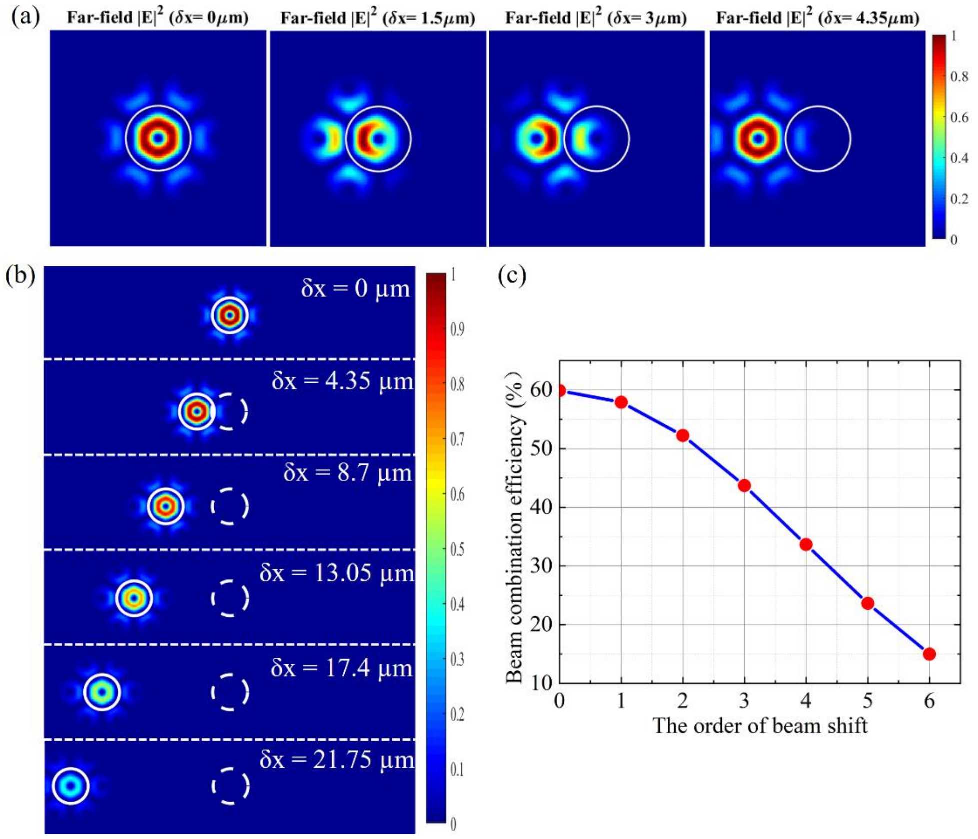

Supplementary Figure 5. (a) The calculated far-field intensity profiles vs fibre positions in the x-direction. (b) Laterally shifted OV beams vs the fibre position (white dotted circle line denotes the position of the unshifted OV beam). (c) Calculated beam combination efficiency of the shifted OV beams.

Supplementary Reference

1. R. Bruning, P. Gelszinnis, C. Schulze, D. Flamm, and M. Duparre, "Comparative analysis of numerical methods for the mode analysis of laser beams," Applied Optics **52**, 7769-7777 (2013).
